# Too Lean

**Published:** 1882-12

**Authors:** 


### TOO LEAN.

IN the November number of the Journal, we endeavored to show how excessive weight of the body might be reduced without danger to health. We have been asked why we did not give instructions for increasing the weight of persons who think themselves *too lean*.

A little reflection will enable one to see that as the causes of these opposite conditions are as unlike as possible, so must be the treatment. To reduce flesh we have simply to reduce the quantity and quality of the food consumed, and when this is done judiciously, the general health of the patient is more likely to be improved than impaired. But were a lean man to attempt to increase his weight by gorging himself with rich food, he would soon find his object defeated, and himself a dyspeptic, and more emaciated than ever. We can not force the body to receive and assimilate more food than it requires ; but there is often a way to increase its requirements, and to thus safely increase the weight of the body, and the general health. A serious obstacle has frequently to be encountered from the fact that *lean* persons are usually nervous and restless ; and these are conditions unfavorable to increase of flesh. This is true of all animals; when it is desired to fatten an animal his range is confined to narrow limits; the food given him is nutritious rather than bulky; he has comfortable quarters, and sleep and quiet are encouraged.

Men are animals, and the same general rules, for the same purpose will apply to them, provided the general health is good. Lazy persons are seldom thin. If, however, emaciation depends on ill health, a different treatment is required. Instead of quiet the patient must have regular exercise in the open air ; horse-back riding if possible, and whatever else that promises improvement of the general health; when emaciation is the result of disease of the lungs, if not too far advanced, this treatment, steadily pursued, often results in permanent cure. Indeed many physicians can refer to persons thus restored to health, who were at one time supposed to be incurable. It has been claimed—and it is probably true—that fresh rich milk is the best of all foods for producing fat. It is true that if a person who is inclined to be fleshy eats bread and milk regularly every day, and particularly in the evening, he will soon assume Aldermanic proportions. Milk is the natural food of man. It is nutritious, and not so liable as other foods to impair digestion.

Sometimes one's mental condition has more to do with bodily health than anything else. As a rule, fat persons are jolly and laughter-loving. Lean people are more often fretful, and have their eyes too steadily fixed on the dark side of every problem. If possible cease to fret and strive to "laugh and grow fat."

